# Depletion of Labile Iron Induces Replication Stress and Enhances Responses to Chemoradiation in Non-Small-Cell Lung Cancer

**DOI:** 10.3390/antiox12112005

**Published:** 2023-11-15

**Authors:** Khaliunaa Bayanbold, Mekhla Singhania, Melissa A. Fath, Charles C. Searby, Jeffrey M. Stolwijk, John B. Henrich, Casey F. Pulliam, Joshua D. Schoenfeld, Kranti A. Mapuskar, Sei Sho, Joseph M. Caster, Bryan G. Allen, Garry R. Buettner, Maria Spies, Prabhat C. Goswami, Michael S. Petronek, Douglas R. Spitz

**Affiliations:** 1 University of Iowa Hospitals and Clinics, Free Radical and Radiation Biology Program, Department of Radiation Oncology, Holden Comprehensive Cancer Center, Iowa City, IA 52242, USAmelissa-fath@uiowa.edu (M.A.F.); garry-buettner@uiowa.edu (G.R.B.);; 2 University of Iowa Hospitals and Clinics, Department Pediatrics, University of Iowa, Iowa City, IA 52242, USA; 3 University of Iowa Hospitals and Clinics, Holden Comprehensive Cancer Center, Department of Biochemistry and Molecular Biology, Iowa City, IA 52242, USA

**Keywords:** ferritin heavy chain, iron chelator, deferoxamine, labile iron pool, cell cycle, replication stress, DNA damage, lung cancer, chemoradiation, holo-transferrin

## Abstract

The intracellular redox-active labile iron pool (LIP) is weakly chelated and available for integration into the iron metalloproteins that are involved in diverse cellular processes, including cancer cell-specific metabolic oxidative stress. Abnormal iron metabolism and elevated LIP levels are linked to the poor survival of lung cancer patients, yet the underlying mechanisms remain unclear. Depletion of the LIP in non-small-cell lung cancer cell lines using the doxycycline-inducible overexpression of the ferritin heavy chain (Ft-H) (H1299 and H292), or treatment with deferoxamine (DFO) (H1299 and A549), inhibited cell growth and decreased clonogenic survival. The Ft-H overexpression-induced inhibition of H1299 and H292 cell growth was also accompanied by a significant delay in transit through the S-phase. In addition, both Ft-H overexpression and DFO in H1299 resulted in increased single- and double-strand DNA breaks, supporting the involvement of replication stress in the response to LIP depletion. The Ft-H and DFO treatment also sensitized H1299 to VE-821, an inhibitor of ataxia telangiectasis and Rad2-related (ATR) kinase, highlighting the potential of LIP depletion, combined with DNA damage response modifiers, to alter lung cancer cell responses. In contrast, only DFO treatment effectively reduced the LIP, clonogenic survival, cell growth, and sensitivity to VE-821 in A549 non-small-cell lung cancer cells. Importantly, the Ft-H and DFO sensitized both H1299 and A549 to chemoradiation in vitro, and Ft-H overexpression increased the efficacy of chemoradiation in vivo in H1299. These results support the hypothesis that the depletion of the LIP can induce genomic instability, cell death, and potentiate therapeutic responses to chemoradiation in NSCLC.

## 1. Introduction

Iron is a critical co-factor in a wide array of cellular processes, including DNA metabolism, as various Fe-S-containing enzymes play essential roles in maintaining high-fidelity DNA replication [1,2,3]. Cancer cells frequently exhibit higher concentrations of labile iron than their normal tissue counterparts [4,5]. Thus, it has been hypothesized that an increased iron content is required by tumor cells to maintain the high proliferative demands associated with neoplastic transformation.

Lung cancer remains the deadliest cancer in both men and women, with an estimated 130,180 new deaths (21% of all total cancer-related deaths) in 2022 [6]; thus, the development of new therapeutic strategies and targets remains essential for improving patient outcomes. Iron has been shown to preferentially accumulate in lung cancer tissue, as these tumors have a phenotype characterized by increased iron import through the transferrin receptor (TfR), altered iron regulatory protein (IRP1/2) activity, and decreased iron export through ferroportin [4,7,8,9]. Therefore, a larger labile iron pool (LIP) and the aberrant expression of iron metabolic genes are often associated with increased lung cancer risk and poor survival [5,10,11,12,13,14]. In addition, serum ferritin has been proposed as a clinical disease marker, but its intracellular and biochemical effects are understudied [15]. Chelating intracellular iron has also demonstrated therapeutic potential in NSCLC, as deferoxamine (DFO) was shown to overcome the cisplatin resistance of A549 non-small-cell lung cancer (NSCLC) cells [16]. Thus, iron metabolism may represent a critical vulnerability that may be exploited therapeutically [4,5,12,17,18]. 

In the present study, the effects of modulating cancer cell iron metabolism in NSCLC cells with different genetic backgrounds was studied using a doxycycline-inducible ferritin heavy chain (Ft-H) overexpression model system, and the clinically relevant iron chelator, DFO. As the primary iron storage enzyme, ferritin consists of multimeric complexes with two distinct subunits: a heavy and a light chain. The Ft-H contains ferroxidase activity and enhances iron sequestration by converting Fe^2+^ into Fe^3+^ for storage in the ferritin protein complexes. Canonically, the Ft-H has been shown to bind iron to mitigate Fenton chemistry and subsequent oxidative damage [19]. Moreover, the Ft-H has been shown to have an enhanced expression as a response to oxidative stress [20]. In this context, the doxycycline-inducible Ft-H overexpression and DFO chelator model systems were used to test the hypothesis that iron availability is critical to maintaining genomic integrity during NSCLC cell growth, as well as a target for sensitizing NSCLC cells to DNA damage repair inhibitors and conventional chemoradiation therapies. 

## 2. Materials and Methods

### 2.1. Cell Culture and Growth Curve

H1299, H292, and A549 were obtained from ATCC (CRL-5803; CRL-1848: CCL-185) and represent non-small-cell lung cancer cell lines. All cells were grown in RPMI media with 10% Fetal Bovine Serum (FBS) in an incubator at 37 °C, 5% CO_2_, and ambient O_2_. All cell lines were utilized before passage 15 and treated in an exponential growth phase at 70–80% confluence. To produce aggressively growing variants of H1299 for use in xenograft models systems, 1 × 10^6^ H1299 cells were injected subcutaneously in the right, rear flank of a female 4–6-week-old athymic nude mice (Foxn1nu/Foxn1nu) purchased from Envigo (previously Harlan Laboratories). A 1000 mm^3^ tumor was removed, washed in sterile PBS, minced in DMEM + 10% FBS + PS containing collagenase/hyaluronidase (Stemcell #07912) and DNase (Roche 11284932001), and incubated overnight at 37 °C in a 5% CO_2_ incubator. The supernatant with digested cells was aspirated and transferred to a 60 mm dish in fresh complete media (DMEM + 10% FBS + PS), and incubated at 37 °C with a media change after 24 h. The cells were allowed to grow to confluence and the cells were passaged 5 more times, every 3–4 days, to allow for any fibroblasts to be depleted from the population. The cells were then renamed H1299T to indicate the passage of H1299 as a tumor through the mice for re-isolation. The H1299T cells were then confirmed to be of H1299 origin by the ATCC Cell Line Authentication Service STR profile report, which showed a 92% match with their data base profile; they were tested for mycoplasma, and then frozen for experimentation. When 1 × 10^6^ H1299T and H1299 cell lines were injected into the nude mice, the H1299T showed 100% (12/12) tumor take versus a 12% tumor take for the H1299 (1/8). 

### 2.2. Development of Ft-H Overexpression Model

Briefly, ferritin heavy chain 1 (Ft-H)-overexpressing cell lines (H1299, H1299T, A549, and H292) were constructed using PCR amplification of a full-length Ft-H from a human liver cDNA library (Clontech). The PCR product and pTRIPZ vector were cut with AgeI and MluI, ligated using T4 DNA ligase, and then transformed into STLB3 competent cells from Invitrogen; the plasmid was extracted and sequenced. The Ft-H plasmid was transfected into 293FT cells using the helper vectors psPAX2 and pCMV-VSV-G (Addgene) to produce lentivirus, which was then used to transduce the H1299T cells, and clones were selected with puromycin, as described in [21]. The clones were expanded, characterized for Ft-H expression following treatment with 1 µg mL^−1^ doxycycline for 48 h, and clones (H1299T Ft-H C11, H1299 Ft-H C4, A549 Ft-H C3, and H292 Ft-H C2) were chosen for further experimentation, as described in [21]. All the NSCLC cell lines, and the parental and doxycycline-inducible Ft-H-overexpressing cells, were grown in RPMI 1640 medium supplemented with 10% fetal bovine serum (FBS). All the cells were cultured at 37 °C in 5% CO_2_, and either 4% O_2_ or 21% O_2_. Ft-H protein expression was temporally induced with 1 µg mL^−1^ doxycycline treatment over 1–7 days. For the iron supplementing experiments, 50–200 µM human hTf (catalog #T3309, Sigma-Aldrich, St. Louis, MO, USA) was added to the cell culture media combined with doxycycline (catalog #195044, MP Biomedicals, LLC, Sydney, NSW, Australia). The following drug treatments were used in this study: deferoxamine (catalog #D9533, Sigma-Aldrich), hydroxyurea (HU) (catalog #C20122850, USBiological life sciences, Salem, MA, USA), VE-821 (catalog #S8007, Selleck Chemicals, Pittsburgh, PA, USA), cisplatin (catalog #NDC 16729-288-38), and etoposide (catalog #NDC 16729-114-31). For the growth curve experiments, the parental and Ft-H-overexpressing colonies of H1299 and A549 cells were incubated with ±1 µg mL^−1^ doxycycline and ±hTf for 0–7 days. The exponentially growing cells of each group at day 3, day 5, and day 7 were collected and counted. Cell numbers vs. corresponding days are plotted as an X, Y graph, and the cell growth rate between treatment groups was analyzed using GraphPad prism 9 software. 

### 2.3. Clonogenic Survival Assay

The treated cells were trypsinized, collected, and counted using a Beckman Coulter Counter. For clonogenic survival analysis, approximately 200–300 cells from each experimental plate were seeded into 6-well cell culture plates and allowed to incubate at 37 °C in either 4% O_2_ or 21% O_2_ for 7–10 days. Once colonies were formed, they were fixed with 70% ethanol and stained with Coomassie blue reagent. Colonies were considered as more than 50 cells, and were counted and plotted to calculate plating efficiency, where plating efficiency is defined as:

$$Plating\;efficiency\left(\%\right)=\left(\frac{number\;of\;colonies\;counted}{number\;of\;cells\;plated}\right)\times 100$$

The plating efficiencies of the experimental groups were normalized to the control group to calculate normalized survival fractions. For the iron rescue experiment, treatment groups of parental cells ± dox, Ft-H-overexpressing colonies ± dox, and their combination with ± Tf were cultured over 48 h. To test the effects on ATR sensitization, either the Ft-H was overexpressed in both the H1299 and A549 cells for 24 h, or both parental cells were treated with 100 µM DFO ± VE-821 drugs overnight. For the radio-sensitization experiments, iron was chelated by Ft-H and DFO in both cells and was followed by sequential exposure to 2–8 Gy at 37 °C in 21% O_2_. Additionally, the cells were treated as a monolayer with ionizing radiation using a cesium radiation source. For the radio–chemotherapy sensitization experiments, following Ft-H overexpression and DFO treatment, the cells were sequentially exposed to a 1 and 3 h chemotherapy combination of 0.5 µM etoposide and 4 µM cisplatin at 37 °C in 4% O_2_, and then 2 Gy IR. 

### 2.4. Western Blotting

Following the associated treatments, the exponentially growing cells were lysed with RIPA and/or DETAPAC buffer containing phosphatase and protease inhibitors, and the cell proteins were extracted. After removal of the cellular debris, a Pierce^TM^ BCA Protein Assay Kit (Thermo Scientific, Rockford, IL 61101, USA) was used to detect the protein concentration on the cleared lysate. Accordingly, 20 µg protein samples were electrophoresed on an 8–14% SDS-PAGE at 80 V for 1.5 h. The separated proteins, based on their molecular weights, were transferred onto a PVDF membrane (Millipore, Billerica, CA, USA), and non-specific bindings were blocked using 5% non-fat dry milk in PBS-Tween (0.2%) solution for 1 h at room temperature. The membranes were incubated with primary antibodies overnight at 4 °C against Ft-H (1:1000; Abcam, 77127, Cambridge, UK) and TfR-1 (1:2000; Life Technology, 136800, Carlsbad, CA, USA). GAPDH (1:5000; Cell Signaling, 5174S, Danvers, MA, USA) and β-tubulin (1:2000; DSHB, E7, Iowa City, IA, USA) were used as loading controls. After 3 × 5 min PBS-Tween washes, the membranes were incubated with corresponding secondary antibodies (1:2000; Sigma-Aldrich, St. Louis, MO, USA), diluted in 5% milk (1:2000) for 1 h at room temperature. The washed membranes were incubated with Super Signal West Pico Chemiluminescent Substrate (Thermo Scientific, Rockford, IL, USA) and exposed to CareStream BioMax MR Film (CareStream Health, Rochester, NY, USA) to visualize the corresponding bands.

### 2.5. Calcein-AM Cytosolic Labile Iron Assay

The labile iron pool (LIP) cellular changes were estimated using the calcein-AM method, as previously described with some modifications [22,23]. Calcein-AM is a “turn-off” probe, where the fluorescence is quenched following iron binding; thus, the iron content of the cell exhibits an inverse relationship to the calcein fluorescence. 

The treatment groups included 48 h ± doxycycline to overexpress Ft-H, and hTf to supplement the iron in the H1299 and A549 cells. The control groups included cells incubated with 40 µM ferric ammonium sulfate (FAS) for 3 h as the positive control, and 100 µM DFO for 3 h as the negative control. Around 2 × 10^6^ cells from each group were washed with DPBS and trypsinized, and cell pellets were collected using centrifugation at 1200 rpm for 5 min. Then, the cell pellets were resuspended in 500 nM calcein-AM diluted in DPBS+HEPES solution, and incubated for 15–20 min at 37 °C. The extracellular calcein-AM was removed using centrifugation and the cells were re-suspended in DPBS. The cell suspension was divided into two groups, with one group incubated with the membrane-permeant intracellular Fe^2+^ chelator 2,2′-bipyridine (BIP, 100 µM) for 15 min at room temperature to provide iron specificity. Samples were immediately run under a UV-LSR violet filter with 515/15 emission. Data analysis was performed using FlowJo V10 software. Relative LIP measures were calculated using the following equation:

$$Relative\;LIP={MFI}_{BIP+}-{MFI}_{BIP-}$$

where MFI_BIP+_ is the mean fluorescence intensity (MFI) with BIP added, and MFI_BIP-_ represents samples measured without BIP. The groups were normalized to the untreated controls.

### 2.6. mRNA Expression Analysis and qPCR

To measure the iron-related genes’ mRNA expression levels following Ft-H overexpression in the H1299 and A549 cells, the cells treated with ±dox over 48 h were collected for RNA extraction using TRIzol^®^ Reagent (ambion^®^ by Life technologies, Carlsbad, CA 92008, USA), following the manufacturer’s recommendations. The purified RNA concentration was measured using NanoDrop^®^ ND-1000 and reverse transcribed using High-Capacity cDNA Reverse Transcription Kit (appliedbiosystems by Thermo Fisher Scientific (Waltham, MA, USA) #57812). The cDNA samples were then used for a real-time PCR using PowerSYBR^®^ Green PCR Master Mix (appliedbiosystems by Thermo Fisher Scientific, Ref 4367659) and QuantStudio 3 (appliedbiosystems by Thermo Fisher Scientific) cycler, based on a previous protocol [24]. Each gene’s mRNA expression levels were quantified and normalized to internal control expressions of GAPDH and S18. The experimentally used Primers and corresponding PrimerBank IDs are as follows: TfR-1 189458816c1, IREB2 133925807c1, SLC40A1 187607385c1, GAPDH 378404907c1, and S18 186928836c1.

### 2.7. Cell Cycle Flowcytometry Analysis

A total of 1 × 10^5^ cells were seeded in 60 mm dishes ± 1 µg mL^−1^ dox to overexpress Ft-H and ± Tf. The cells’ pellets were collected and fixed with 70% cold ethanol. The fixed cells were stained with 1 µg mL^−1^ propidium iodide (PI) (catalog #P4170-25MG, Sigma-Aldrich) and the cell cycle distribution was analyzed with a UV-LSR flow cytometer, by measuring the red fluorescence of the PI-stained DNA content. Cell cycle distribution (%) based on DNA content was calculated using FlowJo V10 software.

### 2.8. BrdU Pulse and Chase Assay

DNA synthesis efficiency and cell movement through the different cell cycle phases were analyzed for the 48 h ± dox H1299 cells using BrdU Pulse and Chase assay protocol, modified from [25,26].

The asynchronized cells were stained with 100 µL BrdU (1 mM stock: Sigma-5-bromo-2-deoxyuridine, B-5002-1 g) and incubated for 30 min at 37 °C in 21% O_2_ to visualize the cells in the DNA replicating stage of S-phase. Then, the BrdU-positive cells were chased with 10 µM cytidine and thymidine, cultured, and collected at 0, 6, and 10 h time points. Harvested live cells were fixed with 70% cold ethanol and suspended in freshly prepared 1 mL 0.2 mg mL^−1^ pepsin dissolved in 2 M HCl, and incubated for 30 min at RT. To neutralize the acid in the solution, 3 mL 0.1 M Borax was added. Then, the cell pellets were collected and washed twice with 0.1% tween-added PBS prior to the addition of a BrdU antibody (1:10; catalog#347580, BD Biosciences, Dubai, United Arab Emirates). Samples were shaken and incubated at room temperature in the dark for 60 min. After two additional wash steps, the cells were incubated with an FITC-conjugated secondary antibody (1:10; catalog#349031, BD Biosciences) for 60 min in a dark room. The pellets were then washed and resuspended in 1 mL PBS containing propidium iodide (35 µg mL^−1^) and RNAse A (1 mg mL^−1^). Following the 1 h incubation in the dark at RT, the cells were run through FACS flow cytometry. Cell gates were created, and the mean fluorescence was measured using FlowJo software. The BrdU-positive undivided cells’ relative movement (RM) was calculated using the equation in Figure S2B,C, to measure the cells’ transit through the S phase. The BrdU-positive G_1_ cells’ fraction (Fr. G_1_^+^) was calculated using the equation in Figure S2B,D, to measure transit through the S phase and G_2_ + M phases. 

### 2.9. DNA Comet Assay

Ft-H was overexpressed in H1299 and A549 cells with doxycycline at 0, 24, 48, and 72 h timepoints. The Ft-H-non-expressing cells were treated with 100 µM DFO, 1 mM HU, and 16 Gy as a positive and negative control for the alkaline and neutral comet assays. Following treatment, cells were collected and processed through a comet assay kit (Catalog#4250-050-K, Trivegen, Gaithersburg, MD, USA) according to the manufacturer’s instructions. To visualize and quantify the comets, the slides were imaged using a BX-61 light microscope (Olympus, Center Valley, PA, USA), and the images were analyzed using CometScore Software (TriTek Corp., Sumerduck, VA, USA). As a DNA damage measurement, the percentage of DNA in the comet tail was calculated and the treatment groups were compared to the non-treated control groups. 

### 2.10. Murine Xenograft Models

Female 4- to 6-week-old athymic nude mice were used for all mouse experiments (IACUC #2031774). An amount of 5 × 10^6^ H1299T cells that overexpress the ferritin heavy chain were injected subcutaneously into the right flank of the mice. Once the tumors were palpable (100 mm^3^), mice in the doxycycline group were treated with 10 mg kg^−1^ doxycycline given intraperitoneally (i.p.), and the control mice were treated with an equivalent dose of NaCl. Following this, the mice in the doxycycline group were fed with chow infused with 1 g kg^−1^ doxycycline for the specified duration of the experiment. The special chow was purchased from Envigo (2018, 1000) (TD.05298). Mice in the control group were fed with normal chow. Tumors were measured every day, or every other day, with Vernier calipers to calculate the tumor volume:

$$Tumor\;volume\left({mm}^{3}\right)=length(mm)\times \frac{{width(mm)}^{2}}{2}$$

Mice were euthanized when the tumor length exceeded 1.5 cm in any dimension for two consecutive days. Tumor tissue was flash frozen in liquid nitrogen and stored in a −80 °C freezer for further analysis. Animals in the treatment groups received all drugs by i.p. injections. In the morning, they received 2 Gy radiation on their flank tumor specifically, given in 10 fx of 2 Gy each, over a period of 2 weeks. In the afternoon, they received 2 mg kg^−1^ of cisplatin, and in the early evening they received 6 mg kg^−1^ of etoposide, and both these chemotherapy drugs were given once a week for 4 weeks. 

### 2.11. Statistical Methods

All analyses were performed using GraphPad Prism^®^ (GraphPad Software, Inc., San Diego, CA, USA). For two groups analysis, an unpaired two-tailed Student’s t-test was utilized. To analyze differences between three or more groups, a one-way (ANOVA) analysis with Tukey’s multiple comparison test was used. For all clonogenic survival curves, the log-rank Mantel–Cox test was used. Experiments were performed with a minimum of 2–3 technical replicates and were repeated on 2 or more separate days to validate the results. For each experiment, *n* represents the number of individual biological replicates. Data are expressed as the mean ± standard deviation (SD), and significance is indicated as follows: *p* < 0.05 (*), *p* < 0.01 (**), *p* < 0.001 (***), and *p* < 0.0001 (****), respectively. 

## 3. Results

### 3.1. Ft-H Overexpression Depletes Labile Iron and Alters Iron Metabolism

**Figure 1:**
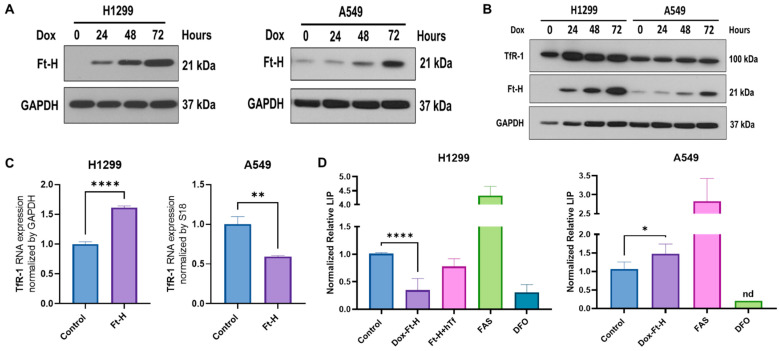
Ft-H overexpression alters intracellular labile iron and induces changes in iron metabolic proteins. (**A**) Ft-H was overexpressed in H1299 Ft-H C4 and A549 Ft-H C3 NSCLC lines following a 1 µg mL^−1^ doxycycline treatment and Ft-H overexpression levels at 24, 48, and 72 h were validated using Western blots. GAPDH was used as the loading control for all the Western blots. (**B**) Ft-H was overexpressed in H1299 and A549 cells following a 1 µg mL^−1^ doxycycline treatment at 24, 48, and 72 h time points, and the expression of Ft-H and transferrin receptor 1 (TfR-1) were detected using Western blots. (**C**) mRNA levels of TfR-1 were detected using qPCR in the Ft-H-non-expressing control cells and the 48 h doxycycline-treated Ft-H-overexpressing cells, and each gene expression level was normalized to GAPDH and 18S RNA. (**D**) LIP was measured at 48 h of Ft-H overexpression in both cell lines using the calcein-AM probe and flow cytometry. Cells treated with 40 µM ferrous ammonium sulfate (FAS) and 100 µM DFO for 3 h were used as the positive and negative control, respectively. Normalized LIP was measured using calcein-AM. An amount of 200 µg mL^−1^ Transferrin (hTf) was used to supplement the cell culture over the same time as the Ft-H overexpression in the H1299 cells. Data are represented as mean ± SEM. *, ** indicate significant differences between the control and treated groups (*n* = 3; * *p* < 0.05, ** *p* < 0.01, and **** *p* < 0.0001); nd refers to non-detectable.

A vector-inducing doxycycline (dox)-inducible Ft-H expression was introduced into NSCLC H1299 and A549 cell lines using lentiviral transduction. Ft-H overexpression following 24–72 h of the dox treatment in the H1299 and A549 cells was confirmed using Western blot (Figure 1A). To assess the functional consequence of Ft-H overexpression, changes in the iron metabolic proteins levels and labile iron pools (LIP) were characterized following Ft-H overexpression (Figure 1B–D). 

The time-dependent Ft-H overexpression consistently increased the transferrin receptor 1 (TfR-1) immunoreactive protein level in the H1299 cells, but not in the A549 cells (Figure 1B). Similarly, the mRNA levels of TfR-1 were upregulated following the 48 h Ft-H induction in the H1299 cells, while they were decreased in the A549 cells (Figure 1C). 

Further functional validation of the Ft-H overexpression was performed using the iron-sensitive calcein-AM probe. Consistent with its function as an iron storage protein, 48 h of Ft-H overexpression significantly decreased the LIP by 70% in the H1299 cells (Figure 1D). Interestingly, the Ft-H significantly increased the LIP by 50% in the A549 cells. Consistent with the overexpression of TfR-1, cell culture supplementation with holo-transferrin (hTf) restored the LIP depletion caused by the Ft-H in the H1299 cells (Figure 1D). Moreover, DFO significantly decreased the LIP of both cell lines, and ferrous ammonium sulfate (FAS) increased the labile iron in both cell lines. Thus, Ft-H overexpression and DFO represent useful model systems for testing the downstream cellular effects of LIP depletion. These findings also underscore the cell-type specificity of iron metabolic regulation. 

### 3.2. LIP Depletion Impairs Cell Growth and Survival by Delaying S-Phase Transit

**Figure 2:**
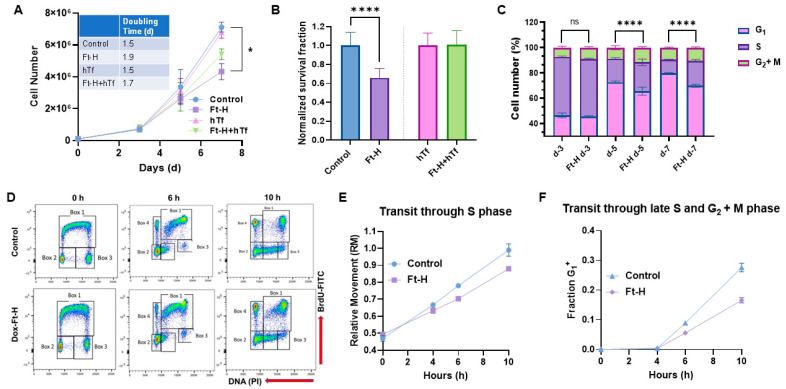
Iron depletion inhibits H1299 Ft-H C4 tumor cell growth by delaying cell cycle progression through the S-phase. (**A**) Ft-H was overexpressed in H1299 Ft-H C4 cells with 1 µg mL^−1^ doxycycline treatment over 7 d. The cell numbers were counted at 3, 5, and 7 d of exponential growth of the cells, and plotted as a cell-growth curve. An amount of 200 µg mL^−1^ hTf was used to supplement the control and Ft-H-expressing H1299 cells with iron over the same time as the doxycycline treatment. A nonlinear regression exponential growth equation was used to calculate the doubling time (d). R^2^ values > 0.94; *n* = 3 separate experiments; error bars are SEM; * indicates significantly different doubling times. (**B**) Ft-H was overexpressed in H1299 Ft-H C4 cells over 48 h and clonogenic survival was measured. The same dose of hTf (200 µg mL^−1^ hTf) was used to supplement the control, Ft-H-overexpressed, and iron-depleted cells with iron, and the clonogenic survival of each treatment was measured. (**C**) The asynchronized control and Ft-H-overexpressing cells of H1299 Ft-H C4 over 3, 5, and 7 days (d-3, d-5, and d-7) were collected, fixed with ethanol, and stained with propidium iodide (PI) to determine the DNA content and cell cycle phase distributions (G_1_, S, and G_2_ + M). (**D**) The control and 48 h Ft-H-overexpressing cells of H1299 Ft-H C4 were incubated with BrdU at time 0 and chased with thymidine for an additional 6 and 10 h. Scatter plot histograms of each time point were displayed as BrdU-FITC fluorescence (Y-axis) and PI-DNA content (X-axis). Cell cycle progression was calculated following the description found in [25] which is also described in Figure S5. (**E**) The relative movement (RM) was calculated to measure the cells progression through the S-phase. (**F**) The fraction of cells in the G_1_ of the daughter generation was calculated to measure the progression of the BrdU-positive S-phase cells through the G_2_ and M phases. ns refers to non-significant (*n* = 3; * *p* < 0.05, **** *p* < 0.0001).

Intracellular iron is essential for cell proliferation and survival; thus, the effects of Ft-H overexpression and LIP depletion on H1299 cell proliferation and survival were evaluated. Ft-H overexpression significantly slows cell proliferation and impairs clonogenic cell survival in H1299 cells (Figure 2A,B). The proliferative effects were partially reversed, and the cytotoxic effects were completely reversed with hTf supplementation, supporting the hypothesis that impaired cell growth and survival results from the depletion of the LIP associated with Ft-H overexpression. This effect was independent of doxycycline toxicity, as doxycycline did not alter proliferation or clonogenic cell survival in the parental H1299 cells (Figure S1A,B). In contrast to H1299, Ft-H overexpression in A549 cells did not induce changes in cell growth or clonogenic survival (Figure S2A,B). Overall, Ft-H overexpression only appeared to decrease the LIP, inhibit cell growth, and clonogenic cell killing in H1299 NSCLC.

The cell cycle distribution was determined to further evaluate the underlying mechanisms of how LIP depletion results in the impairment of cell proliferation and clonogenic survival in H1299 cells. The time-dependent overexpression of the Ft-H led to the accumulation of cells in the S and G_2_ + M phases, and a reduction in the cells in the G_1_ phase by day 5, as detected using propidium iodide staining and flow cytometry analysis (Figure 2C). These cell cycle changes and inhibition of cell growth were also observed in the Ft-H-overexpressing H292 cells (Figure S3A,B). A BrdU pulse–chase assay was employed to better understand the impact of the Ft-H overexpression-induced decreases in the LIP on the transit of cells through each phase of the cell cycle. The cells synthesizing DNA at the time of labeling (0 h) and S phase (Box 1) incorporate BrdU, while both the G_1_ (Box 2) and G_2_ (Box 3) phases are indicated as BrdU negative at time 0 h (Figure 2D and Figure S5). The representative flow cytometry plots (Figure 2D) show cell movements through the different cell cycle phases over 6 h and 10 h time periods post BrdU labeling in the control and Ft-H-overexpressing H1299 cells. Another group of cells was identified (Box 4) as BrdU-positive cells with G_1_ DNA content that completed cell division (Figure 2D and Figure S5). The relative movement (RM) of the BrdU-positive S phase cells was used as the measure of transit through the S phase, while the fraction (Fr. G_1_^+^) of BrdU-positive G_1_ cells (Box 4) was measured to analyze the transit through the S phase and G_2_ and M phases (Figure 2D and Figure S5). Consistent with the observed accumulation of S-phase cells, Ft-H overexpression caused a delay in transit through the S-phase based on the lower relative movement (RM) curve (Figure 2E), and a delayed transit through the late S into the G_2_ + M phase, indicated by the slower accumulation of the G_1_^+^ fraction of the cells (Figure 2F). Thus, LIP depletion by Ft-H overexpression appears to inhibit cell growth by delaying transit through the S-phase of the cell cycle and DNA synthesis, indicating possible replication stress caused by the depletion of the LIP. 

### 3.3. Depletion of Intracellular Iron Induces DNA Damage in NSCLC

**Figure 3:**
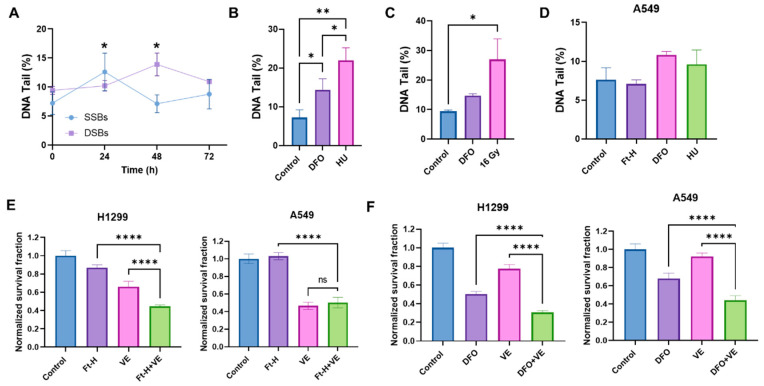
Depletion of labile iron promotes DNA damage. (**A**) Ft-H overexpression was induced over 24, 48, and 72 h in H1299 Ft-H C4 cells with doxycycline. Alkaline and neutral comet assays were performed to measure SSBs and DSBs, respectively. The percentage of DNA in the comet tail (DNA tail %), out of 100%, was measured for each treatment group to determine the SSBs and DSBs using CometScore 2.0 software. (**B**) The alkaline comet assay was used for the SSBs from the H1299 cells treated with 100 µM DFO overnight to deplete the intracellular iron with 2 µM hydroxyurea (HU) overnight as a positive control. (**C**) The neutral comet assay was used for the DSBs from the 100 µM DFO overnight-treated H1299 cells with 16 Gy as a positive control. (**D**) The alkaline comet assays were used for the A549 Ft-H C3 treated with doxycycline for 48 h to over express Ft-H, 100 µM DFO overnight, or 2 µM HU overnight. (**E**) The H1299 Ft-H C4 cells were treated with 5 µM and the A549 Ft-H C3 cells were treated with 10 µM ATR inhibitor (VE-821) over 24 h, alone and in combination with the 24 h Ft-H expression. The survival fraction of each cell group, with and without VE, was determined using a clonogenic assay and normalized to the Ft-H-non-expressing control cells. (**F**) Both cell lines were treated as in (**E**), with 100 µM DFO ± ATR inhibitor (VE-821) overnight. Clonogenic survival was assayed, and the DFO- and VE-alone treated groups, and the combination of DFO and VE treated groups, were normalized to the respective untreated control. ns refers to non-significant (*n* = 3; * *p* < 0.05, ** *p* < 0.01, and **** *p* < 0.0001).

Based on the previously observed S-phase accumulation and DNA synthesis delay, it was hypothesized that the Ft-H induces an impairment in cell survival, resulting from DNA replication stress and DNA damage [27,28]. Consistent with this hypothesis, the Ft-H caused a time-dependent increase in single-stranded and double-stranded DNA breaks (SSBs and DSBs) in the H1299 cells (Figure 3A). The SSBs induced by the Ft-H peaked at 24 h (+75%) and then decreased to basal levels by 48 h in the H1299, while the DSBs peaked at 48 h (+78%). Furthermore, DFO treatment resulted in both SSBs and DSBs similar to those resulting from the replication stress-inducing agents, hydroxyurea (HU) and 16 Gy radiation, respectively (Figure 3B,C). Furthering the observed cell specificity, these effects were not observed in the A549 cells that were Ft-H overexpressed and, while treatment with DFO was able to promote SSBs, it did not reach statistical significance (Figure 3D). Since replication stress-associated replication fork stalling and breakage activates the ATR DNA damage response [29,30], we hypothesized that Ft-H overexpression would sensitize NSCLC cell lines to ATR inhibition. Consistent with our hypothesis, 24 h Ft-H overexpression enhanced the toxicity associated with the ATR inhibitor VE-821 in H1299 (Figure 3E). This effect was not observed in the A549 cells. DFO enhanced the toxicity of VE-821 in both cell lines (Figure 3F). This is likely the result of the higher stability of the iron DFO complex compared to that of the Ft-H [31], and is consistent with the previous observation that DFO induced an LIP depletion in the A549 cells. Therefore, these data support the hypothesis that the depletion of the LIP may induce replication stress in NSCLC cells that is exacerbated by ATR inhibition. 

### 3.4. Depletion of Intracellular Iron Enhances the Effectiveness of Radiation and Chemotherapy in NSCLC

**Figure 4:**
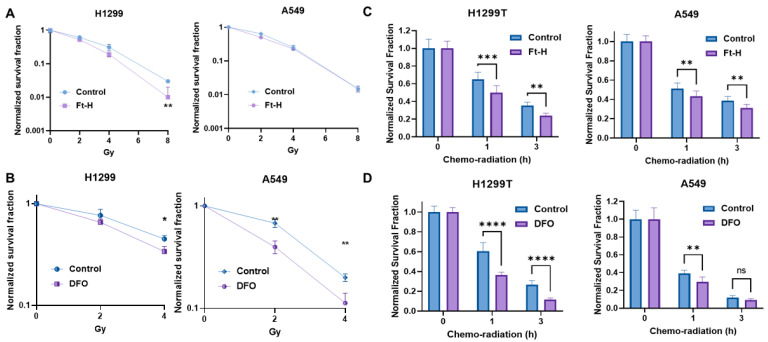
Depletion of labile iron enhances NSCLC sensitivity to chemoradiation in vitro. (**A**) Ft-H was induced for 48 h with 1 µg mL^−1^ doxycycline in both cell lines (H1299T Ft-H C11 and A549 Ft-H C3), and exposed to 2, 4, and 8 Gy radiation. Clonogenic survival was analyzed and compared within the groups of non-Ft-H-expressing and Ft-H-expressing cells, and normalized to the respective, untreated control. (**B**) The parental cells of the H1299 and A549 were treated with 100 µM DFO over 12 h and exposed to 2 and 4 Gy radiation. Clonogenic survival of each radiation dose was normalized to the control or DFO treated cells, respectively. (**C**) Following 48 h doxycycline treatment in the H1299T Ft-H C11 and 72 h in the A549 Ft-H C3, the cells were treated with a combination of 4 μM Cisplatin and 0.5 μM Etoposide for 1 to 3 h, followed by 2 Gy radiation. Throughout drug treatment, cells were cultured in 4% O_2_. Clonogenic survival was determined and compared between the control and Ft-H-expressing cells after chemo-radiation therapy. (**D**) The parental H1299 and A549 were treated with 100 µM DFO over 12 h to chelate intracellular iron and then treated with a combination of chemoradiation as in (**C**), over the same time period at 4% O_2_. Clonogenic survival was determined and compared between the control and DFO-treated cells following the treatment. Error bars represent mean ± SEM for 3 independent experiments, with * *p* < 0.05, ** *p* < 0.01, *** *p* < 0.001, and **** *p* < 0.0001, ns = not significant: using a two-way ANOVA test.

The induction of replication stress is an efficient strategy with which to enhance tumor cell killing and the response to therapy [32,33,34]. Thus, it was hypothesized that LIP depletion could enhance the effectiveness of radiation and chemotherapy. Consistently, Ft-H overexpression sensitizes H1299T cells to ionizing radiation, but not A549 cells (Figure 4A). Alternatively, iron chelation by DFO sensitizes both cells to radiation treatment (Figure 4B). Interestingly, there DFO had a stronger effect on the A549 cells compared to the H1299T cells in terms of iron chelation-induced radio-sensitization.

Ft-H overexpression enhanced clonogenic cell killing with combined radiation and chemotherapy in both the H1299T and A549 cells (Figure 4C). Similarly, DFO sensitized both cells to chemoradiation in vitro (Figure 4D). Overall, iron chelation by either Ft-H overexpression or DFO treatment enhances the clonogenic death of NSCLC cells combined with radiation alone and/or a combination treatment with chemotherapy in vitro.

### 3.5. Ft-H Overexpression Sensitizes NSCLC to Chemoradiation In Vivo

**Figure 5:**
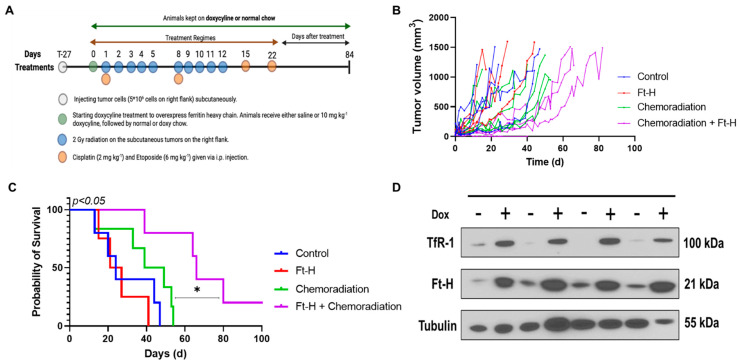
Overexpression of Ft-H sensitizes H1299T xenografts to chemoradiation. (**A**) Schematic representation of H1299T Ft-H C11 tumor chemoradiation treatment protocol in nude mice with Ft-H overexpression induced by doxycycline. (**B**) Tumor volume of each animal in the treatment groups in mm^3^. (**C**) Kaplan–Meier survival plot of H1299T Ft-H C11 tumor-bearing mice after receiving chemoradiation therapy. Mice received 10 mg kg^−1^ doxycycline or an equivalent dose of NaCl given IP, 24 h prior to chemoradiation treatment, and were fed chow containing 1 g kg^−1^ doxycycline for the duration of the experiment. The tumors received a total of 20 Gy radiation to their flank tumor, and this was given in 10 fx of 2 Gy each, over a period of 2 weeks. The animals were treated following radiation with 2 mg kg^−1^ of cisplatin followed by 6 mg kg^−1^ of etoposide, and these chemotherapy drugs were given once a week for 4 weeks. (**D**) Tumor tissue from the groups receiving chemoradiation + doxycycline was collected at euthanasia and homogenized for Western blotting of TfR-1 and Ft-H and compared to chemoradiation alone (*n* = 3; * *p* < 0.05).

A subcutaneous xenograft model system was utilized to test the effects of Ft-H overexpression on H1299T responses to radiation and chemotherapy in vivo using the protocol shown in Figure 5A. Tumor volumes from each treatment group were measured and plotted versus the day of treatment (Figure 5B). The tumor volumes were used to determine survival, and the mice with tumors that measured ≥1.5 cm in any direction on consecutive days were euthanized, as specified in the protocol. The Kaplan–Meier survival curves show that the median overall survival (24 days) for the control and Dox-Ft-H groups were not significantly different. However, Ft-H overexpression slowed tumor growth (*p* = 0.06) and significantly enhanced the therapeutic responses to cisplatin/etoposide + 10 × 2 Gy radiation (median overall survival, 44 d vs. 65 d; *p* < 0.05, Figure 5C). For the in vivo validation of overexpression, four tumors were collected at euthanasia and, similar to the in vitro results shown in Figure 1B, a Western blot confirmed the overexpression of the Ft-H and the associated increase in TfR-1 expression (Figure 5D). These data support the hypothesis that LIP depletion by the overexpression of the Ft-H enhanced the effectiveness of radiation and chemotherapy in vivo. 

## 4. Discussion

It has been hypothesized that increased iron content is an oncometabolite required for cancer growth during its initiation, promotion, and progression [4,35,36,37]. It has previously been reported that NSCLC tumors from patients have higher LIP levels than adjacent normal lung tissue [5]. LIP depletion using chelators has been proposed as a novel therapeutic strategy with which to enhance the efficacy of chemoradiation treatment, but the molecular mechanisms are still unresolved [38,39,40]. The goal of this study was to interrogate these mechanisms by depleting labile iron genetically, using Ft-H overexpression, and pharmacologically, with DFO treatment in NSCLC cells. The current findings indicate that the depletion of the LIP by the overexpression of the Ft-H impaired cell growth and survival in the H1299 and H292 cells, but not in the A549 cells. This may result from differences in their genetic backgrounds, as A549 cells have a robust adaptive response to iron chelation [41], as well as harboring mutations in KRAS, STK11, and KEAP1 that are not found in H1299 and H292 that may give rise to differential iron metabolic regulation. 

In this study, the H1299 and H292 cells were found to be more sensitive to iron depletion-induced stress. The depletion of intracellular iron induced cellular stress that was characterized by the accumulation of cells in the S-phase, in conjunction with the temporal accumulation of both SSBs and DSBs. Interestingly, the initial accumulation of SSBs after 24 h was resolved by 48 h, whereas an accumulation of DSBs were observed. This supports the hypothesis that by impairing the DNA replication machinery through LIP depletion, the SSBs are being fixed in place to form more complex DSBs. These observations are characteristic of the induction of genomic instability and DNA replication stress, as well as being consistent with previous studies that have shown that iron chelation can cause G_1_ and S phase cell arrest, DNA damage, and apoptosis [42]. 

Iron availability is crucial for proper DNA replication, and iron depletion causes replication stress, genome instability, and cell death in mammalian cells [43]. A possible explanation for these observations may relate to the impaired function of nuclear iron–sulfur (Fe-S) clusters following iron limitation. Several key DNA metabolic enzymes (including DNA primase and eukaryotic DNA polymerases, DNA repair helicases, nucleases, glycosylases, demethylases, and ribonucleotide reductase) contain a [4Fe-4S]^2+^ cluster (or a di-ferric center in the case of ribonucleotide reductase) that must be intact for enzymatic activity and high-fidelity DNA replication [1,44]. Consistent with this hypothesis, impaired Fe-S biogenesis has been shown to cause the degradation of [4Fe-4S]^2+^ containing DNA metabolic enzymes, resulting in replication fork collapse, genomic instability, and cell death [45,46,47]. Another potential cell death mechanism that warrants further investigation is apoptosis induction, as both DFO and the Ft-H have previously been shown to induce apoptosis in NSCLC [16,48]. The induction of apoptosis would be consistent with the working hypothesis that intracellular iron serves as a chemical switch between apoptosis and ferroptosis in cancer cells [49]. In addition, iron depletion may also disrupt Fe-dependent cellular oxidative metabolism enzymes, including ETC enzymes, resulting in increased oxidative stress, which may combine with chemoradiation for increased sensitization [50]. Conversely, mitochondrially derived oxidative stress and iron accumulation associated with ferroptosis have been proposed as promoting tumor cell migration associated with disease progression [51,52,53,54,55]. Therefore, the mitigation of iron-catalyzed oxidative distress by the Ft-H may be a contributing factor to its inhibition of tumor growth. Because of the pleiotropic nature of iron’s regulation of cellular metabolism and biological outcomes, each of these potential mechanisms of cell death warrants further consideration in future studies related to Ft-H overexpression.

Consistent with the hypothesis related to replication stress, the overexpression of the Ft-H or treatment with DFO enhanced the toxicity of the ATR inhibitor VE-821. These results are compatible with this hypothesis, as the ATR DNA damage response pathway is activated upon replication fork collapse [56,57]. Thus, targeting the Fe-S biogenesis pathway to promote genomic integrity may be an efficient strategy with which to enhance cancer therapy.

This study also highlights the translational potential of these fundamental observations. Ft-H overexpression or treatment with DFO were shown to enhance cellular radiation responses and radio–chemosensitivity both in vitro and in vivo. These results are consistent with previous studies that have shown that depleting iron can enhance radio-sensitivity through ATR activation [4,7,38,58,59,60]. In addition, these data are consistent with previous results that have shown that DFO enhanced cisplatin sensitivity in A549 cells, a result that was replicated in the context of chemoradiation in the current study [16]. Similar therapeutic sensitization was observed in the A549 cells treated with DFO, but could not be replicated using Ft-H overexpression, likely due to the high stability constant of the ferrioxamine complex (i.e., Fe^3+^-DFO) [61]. Overall, it was observed that LIP depletion can induce replication stress and could be a useful therapeutic approach in NSCLC therapy that warrants further investigation. 

## 5. Conclusions

This study has interrogated the effects of targeting iron metabolism in NSCLC using pharmacologic and unique genetic approaches to uncover the following key points:

- The depletion of labile iron impairs cell growth and survival in NSCLC;
- The overexpression of the Ft-H leads to the accumulation of cells in the S-phase of the cell cycle and increased DNA damage, indicative of DNA replication stress;
- The overexpression of the Ft-H or treatment with DFO enhances the toxicity of ATR inhibition in NSCLC;
- The overexpression of the Ft-H or treatment with DFO enhances radiation and chemotherapy responses in NSCLC in vitro and in vivo.

## Data Availability

The data are contained within the article and are available on request from the corresponding author.

## Supplementary Materials

The following supporting information can be downloaded at: https://www.mdpi.com/article/10.3390/antiox12112005/s1.

## Abbreviations

**DFO**, deferoxamine; **FAS**, ferrous ammonium sulfate; **Ft-H**, ferritin heavy chain; **NSCLC**, non-small-cell lung cancer; **HU**, hydroxyurea; **TfR-1**, transferrin receptor 1.
