# Supplementary material for: Depletion of Labile Iron Induces Replication Stress and Enhances Responses to Chemoradiation in Non-Small-Cell Lung Cancer
Source: Antioxidants (Basel). 2023 Nov 15;12(11):2005. doi: 10.3390/antiox12112005 (PMC10669787; doi:10.3390/antiox12112005)
Supplement: Supplementary file 1 [file antioxidants-12-02005-s001.zip › antioxidants-2687287-SI.pdf]

Supplemental Data for

Depletion of Labile Iron Induces Replication Stress and Enhances Responses to Chemoradiation in Non-Small-Cell Lung Cancer

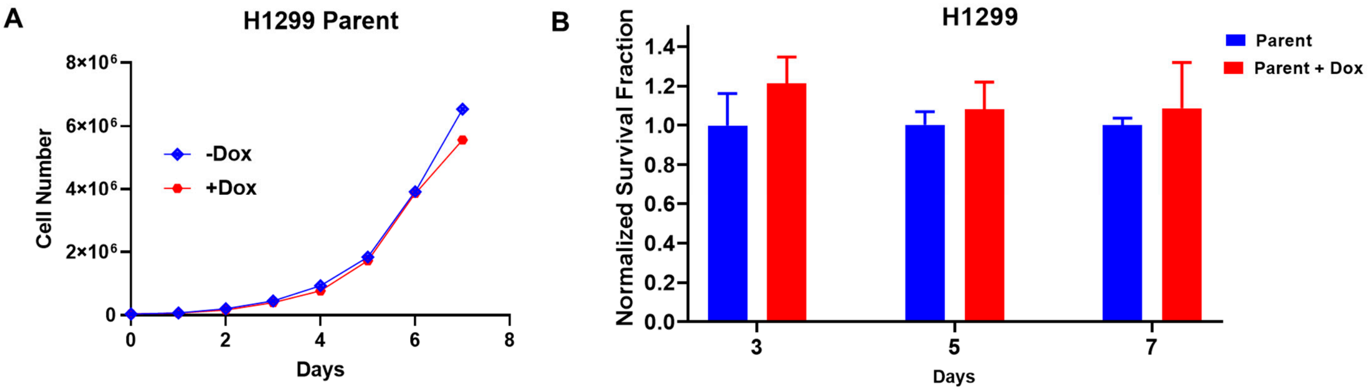

Supplemental Figure S1. **Doxycycline does not interfere with growth curve and clonogenic survival of parental H1299 cells.**

A. H1299 parental cells were treated with 1 μg ml^{-1} doxycycline over 7 days. Cell growth curve was created on ± doxycycline treated H1299 parental cells by counting cell numbers on each day. **B.** Dox inducible Ft-H C4 cells driven H1299 parental cells were treated with ±dox over 3, 5 and 7 days and clonogenic survival rate of each cell group was determined and plotted over each treatment. (*n* = *3*) independent experiments and each experiment has 3 technical replicates.

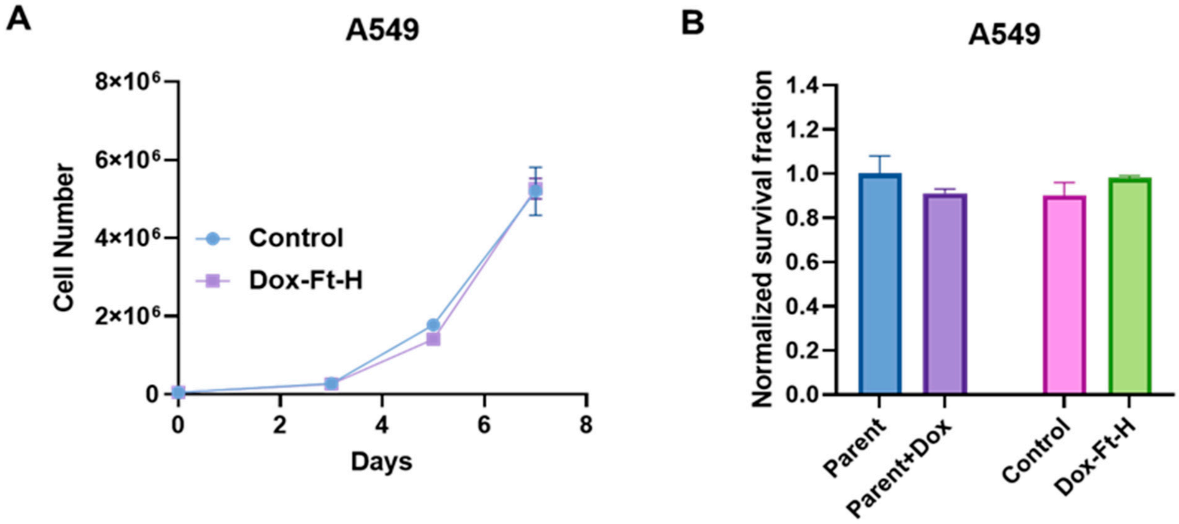

Supplemental Figure S2. Ft-H overexpression does not induce phenotypic changes in A549 cell growth and clonogenic survival.

A. Ft-H was overexpressed in A549 Ft-H C3 with 1 μg ml^{-1} doxycycline treatment over 7 days and cell numbers were counted at 3, 5 and 7 days of exponential growth of the cells and plotted as a cell growth curve. **B.** Doxycycline inducible Ft-H C3 driven A549 parental cells and clonal cells were treated with doxycycline over 48 h to induce Ft-H and clonogenic survival rate was measured in A549 cells. (*n* = *3*) independent experiments and each experiment has 3 technical replicates.

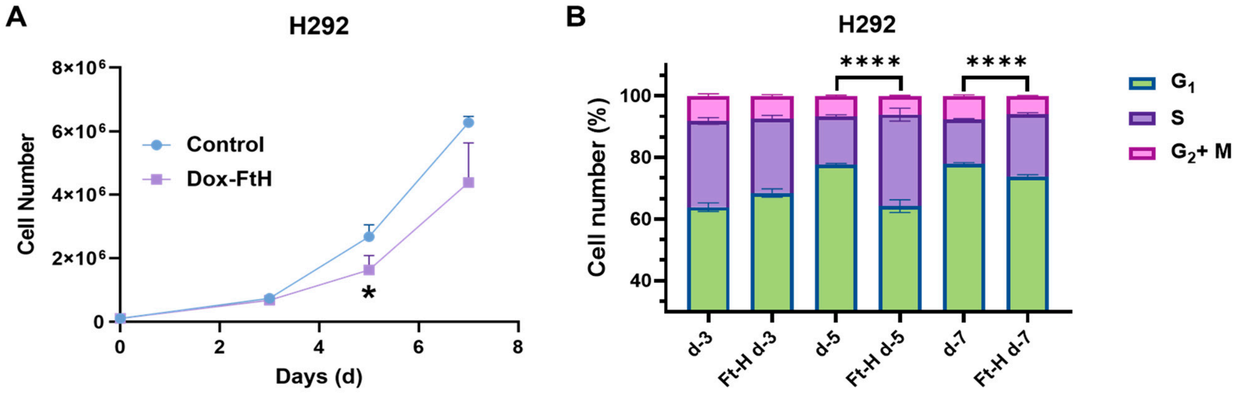

Supplemental Figure S3. Ft-H overexpression in H292 slows growth curve and arrest cell cycle at S phase.
A. Ft-H was stably overexpressed in H292 C2 NSCLC cells over 7 days with 1 µg ml^{-1} doxycycline. Cell numbers were counted at day 3, 5 and 7, and cell growth curve was plotted. **B.** H292 C2 cells collected in **A** were stained with PI and subjected to flowcytometry analysis of DNA content to identify the percentage of cells in the G_1, S, and G_2 + M phases of the cell cycle. S phase cell number (%) within the groups was analyzed statistically. Data are represented as mean ±SEM. *,**** indicates significant differences between control and treated groups (n = 3 *p<0.05 and ****p<0.0001).

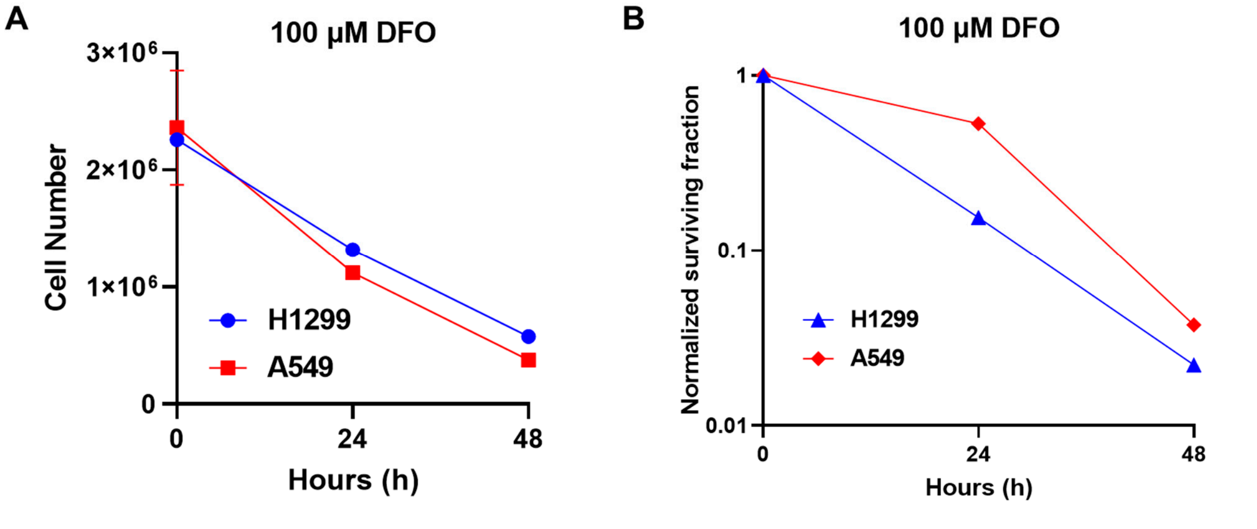

Supplemental Figure S4. DFO inhibits cell growth and clonogenic survival in both H1299 and A549 cells. (**AB)** Exponentially growing H1299 and A549 cells were treated with 100 µM DFO over 24 h and 48 h. Control and DFO treated cells growing in 100 mm culture dishes were taken and counted for cell numbers and clonogenic survival. Corresponding cell growth and clonogenic survival were analyzed and compared between the two cells cell lines. (n = 2) independent experiments and each experiment has 3 technical replicates.

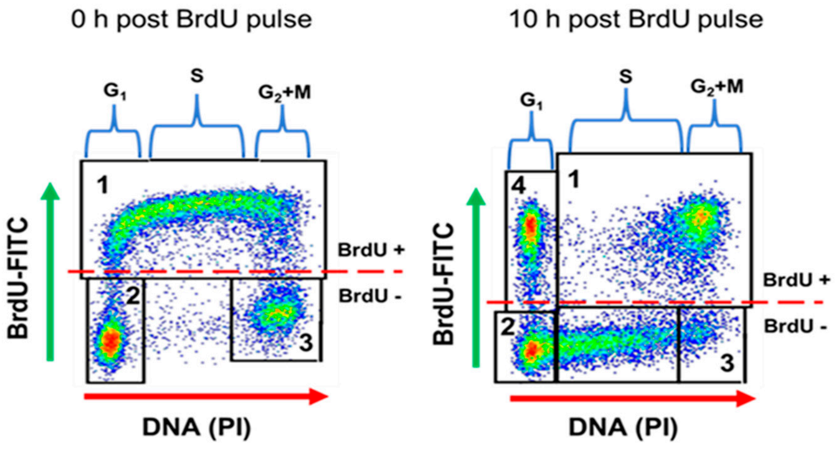

Supplemental Figure S5. Schematic representations of the BrdU histograms that were used to calculate cell cycle progression.

BrdU pulse-chase assay and data analysis were performed following the previously published protocol (Menon et al., 2003).

These various cell populations were used to calculate the relative movement (RM), reflecting the progression of the cell population through S-phase, and the progression of cells through S and $G_2 + M$ phase ($Fr.G_1^+$) using equations 1 and 2:

$$RM = \frac{Box\ 1 - Box\ 2}{Box\ 3 - Box\ 2} \quad (1)$$

where cells in box 1 represent BrdU-positive S-phase cells and cells in boxes 2 and 3 represent BrdU-negative G_1 and G_2 populations, respectively;

$$Fr.G_1^+ = \frac{\left(\frac{Box\ 4}{2}\right)}{Box\ 1 + \left(\frac{Box\ 4}{2}\right)} \quad (2)$$

where cells in box 4 represent BrdU-positive S-phase cells of the parental generation that progressed through G_2 + M phases and reside in the G_1-phase of the daughter generation.
